# Spread of “triad diagnostics” in suspected Shaken Baby Syndrome

**DOI:** 10.1016/j.fsisyn.2026.100660

**Published:** 2026-01-30

**Authors:** Anders Eriksson, Teresa Stachowicz-Stencel, Knut Wester

**Affiliations:** a Department of Clinical Sciences, Forensic Medicine, PO Box 7616, SE-907 37, Umeå University, Umeå, Sweden; b Department of Pediatrics, Hematology and Oncology, Medical University of Gdansk, Dębinki 7 Str., PL-80-211, Gdańsk, Poland; c Department of Clinical Medicine K1, University of Bergen, Bergen, Svingen 42, NO-0196, Oslo, Norway

**Keywords:** Shaken baby syndrome, Abusive head trauma, Circular reasoning, Unreliable diagnostic procedure

## Abstract

We describe here possibly the first two cases of alleged Shaken Baby Syndome (SBS) in Poland, based solely on “triad findings” (encephalopathy symptoms, subdural hemorrhage/SDH, and retinal hemorrhages/RH), but without signs of relevant trauma. Case #1, a 7-week-old infant girl, is suggested to represent a case of rebleeding in a birth-related SDH. In case #2, a 13-week-old infant boy, we claim that the triad findings were related to benign external hydrocephalus (BEH). Unjustified belief that triad findings are always caused by violent shaking may, apart from the obvious legal and social effects, in case #2 also have contributed to delayed adequate treatment of increased intracranial pressure and subsequent signs of permanent brain damage.

We discuss also the traditional SBS hypothesis and its lack of solid scientific evidence, and the uneven geographical acceptance of and belief in this unvalidated hypothesis.

## Highlights

• Two cases of suspected Shaken Baby Syndrome are presented, both misdiagnosed.
• The lack of reliability of the “triad diagnostic method” is emphasized.
• The most common cause of triad findings is probably BEH/BESS.
• Both over- and underdiagnosis of suspected abuse can have disastrous effects.

## 1 Introduction

Shaken Baby Syndrome (SBS) was presented in the 1970's as a hypothesis, suggesting that subdural hemorrhage (SDH) in an infant without signs of trauma could be explained by violent shaking [1,2]. Later also encephalopathy symptoms and retinal hemorrhages (RH) were added – collectively called “the triad”. Despite lack of empirical basis, these findings soon gained widespread acceptance in pediatric medicine as medical and judicial evidence of violent shaking [[3], [4], [5], [6], [7]]. In parallel with the introduction of advanced imaging techniques during the 1980's and 1990's, it was established that in the absence of signs of relevant trauma, triad findings were virtually pathognomonic of SBS. Particularly RH were considered strong evidence of SBS [8].

Despite lack of scientific foundation for this SBS hypothesis, questioning of the hypothesis was scarce, and the use of the simplified “triad diagnostic method” soon spread to many countries, strongly advocated by pediatric proponents. Not until the early 2000's was the reliability of the SBS tenets formally and scientifically questioned through a non-systematic literature review [9], and by the presentation of a non-traumatic hypothesis explaining the triad findings [10]. Both these publications were harshly criticized by the pediatric community and the triad diagnostic method remained. In 2009, SBS was included as a subgroup of the large and heterogeneous group of Abusive Head Trauma (AHT) [11], a step that has complicated rather than facilitated the elucidation of the pathogenesis of triad findings [12].

In 2016, a systematic review from the independent Swedish Health Technology Agency (“SBU”) revealed that the allegedly very high “conclusive evidence” of the triad diagnostic method was merely the result of incorporation bias and circular reasoning [13]. This basic flaw explained also the unreasonably high positive predictive values (PPVs) reported in some systematic reviews (for example [14], [15], [16]) - all assessed to be of low quality due to high risk of bias. In conclusion, the SBU report demonstrated that publications reporting on the diagnostic process of violent shaking of infants were based on assumptions and circular reasoning, not on confirmed shaking. Incorporation bias occurs when a diagnostic test is included in defining the group it is intended to predict – the reference test. Circular reasoning creates artificial associations and makes the diagnostic test appear far more accurate than it is.

The SBU report led to no change in clinical practice nor in the use of the triad as applied in the courts. Instead, strong criticism of the report resulted, and although all criticism was answered, the fundamental errors in the triad diagnostic method - primarily incorporation bias and circular reasoning - were left unrecognized by the pediatric community. In part, this was probably because the serious logical flaw of circular reasoning went unrecognized. Representatives of the Royal College of Paediatrics and Child Health [17] were likewise obtuse to the problem of circular reasoning, along with a number of other “misinterpretations”. These included - but was not limited to - incomprehensible and ignorant criticism of the research question and the search strategy, of the various parts of the applied PIRO, and of alleged “double standards” and “pseudoscience”. The authors also falsely claimed that the triad is never used as diagnostic test in clinical practice and were clearly ignorant of the widely used GRADE method and the concepts of *limited* and *insufficient* scientific evidence - concepts used by HTA agencies for decades. Instead, they clearly revealed their incompetence in this area and did not disclose their clear conflicts of interest. The authors even met relevant counter-questions [18] with an emotional exclamation "*It is NEVER acceptable to shake a baby*" [19], a statement which presumes that shaking is the cause of what is seen, in a true circular fashion. This incompetence boomerangs hard also upon those who assigned the authors, the Royal College of Paediatrics and Child Health.

Another response was the so-called “consensus statement” [7], which maintained that the SBS diagnosis is a strictly “medical conclusion”- although based almost exclusively on publications of very low scientific quality [13]. This “consensus statement” and its advocating review was uncritically accepted by several pediatric and neuroradiological societies [7,20], whereas the legal community published harsh criticism [21,22]. A recent experimental questionnaire study confirmed the value-based nature of the SBS diagnosis, thus contradicting the consensus statement that the diagnosis is a strictly “medical conclusion” [23]. A more recent, lengthy publication, comprising another consensus statement [24], has again given rise to critical flaws revealed by independent researchers [25].

In summary, there remains divisive controversy regarding using the triad as a diagnostic method that can predict SBS/AHT. The fact remains that the traditional SBS hypothesis is unsupported by evidence-based research. Studies claiming to support the hypothesis lack robust reference standards and instead rely on low quality studies with incorporation bias and circular reasoning. This is illustrated by the following presentation of two of the first known Polish infants incorrectly diagnosed according to the tenets of the traditional SBS hypothesis.

## 2 Patients and methods

With the permission of both parental couples, the authors received all relevant documentation for both infants. The presentation of findings and handling of each case is followed by case-specific comments, whereas more general aspects of the SBS/AHT controversy are presented in the Discussion section.

### 2.1 Case #1

This girl was delivered vaginally at term after a slow progress. The postnatal period was uneventful except for severe reflux and cow's milk protein intolerance. At the age of 7 weeks, she suddenly began to cry in the evening, followed by a decreased muscular tone with retained consciousness.

**Fig. 1:**
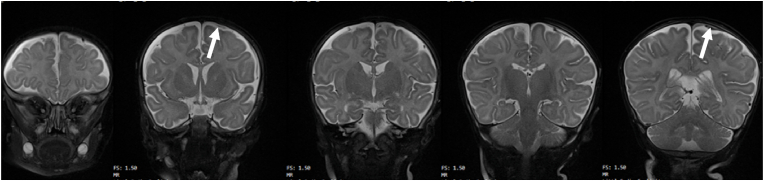
Case #1. Series of coronal MRI scans showing very thin subdural fluid layers (arrows) compatible with chronic SDH (hygroma).

Upon admission to hospital the next morning, all examinations (external, neurological, ophthalmological, and ultrasound, as well as head circumference (HC) development) were normal except for minute skin petechiae on her right thigh and abdomen, and a small bruise by the right eye caused by a baby bottle which had fallen on her. Her general condition was good, and she was sent home the next day. Ten days later a similar incident occurred, the EEG was normal, and MRI revealed thin bilateral, multistage SDH without clinical signs of increased intracranial pressure. The occipital part of the extra-axial hemorrhage

was hyperintense on T1-WI, indicating presence of methemoglobin (Fig. 1), and thus chronicity of the SDH. Despite absence of signs of relevant trauma and a normal skeletal X-ray, she was reported as a suspected case of child abuse to social services, apparently because of numerous RH detected at a second fundoscopy.

When still in hospital, a court order sent the baby to foster care. The parents consulted several international experts, all categorically excluding abuse, and regained custody as the social services had no concerns whatsoever regarding the care or otherwise in the family. At the age of 5 months, complete resolution of SDH and RH was confirmed by MRI and fundoscopy, and she showed a completely normal psychomotor development.

***Comments***: This baby girl had no dramatic symptoms, no signs of relevant trauma and very thin layers of subdural fluid with the appearance of hygroma. Regarding RH, see comments on Case 2 below. Considering the absence of trauma and no concerns whatsoever from the social services, as well as the *insufficient* scientific support for SBS/AHT based on triad findings only, there remains no support for the default diagnosis of child abuse. The findings are compatible with birth-related chronic SDH with re-bleeding [[26], [27], [28]]. The attending physician's report to the social services as suspected child abuse was likely due to routines regarding triad findings and failure to consider other causes.

### 2.2 Case #2

**Fig. 2:**
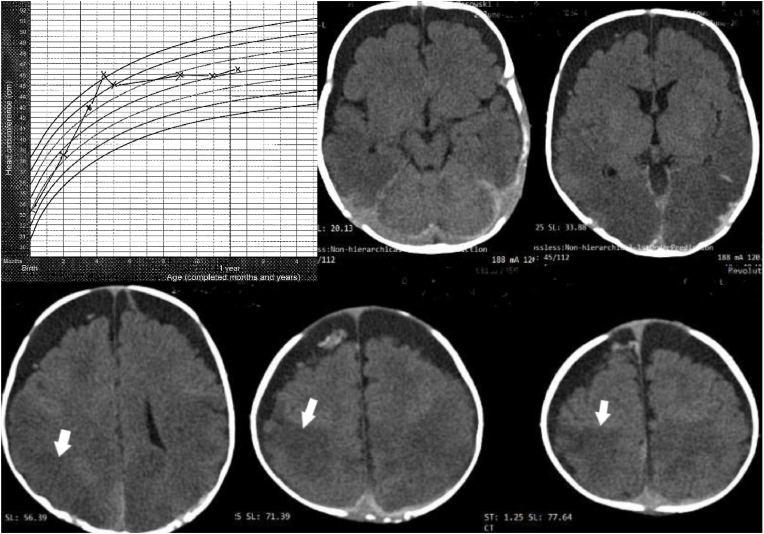
Case #2. Upper left: head circumference (HC) development from birth plotted on the WHO HC chart for boys shows a steep and too rapid initial HC increase – crossing several percentiles during the first four months, indicating hydrocephalus development [23]. Thereafter, there is a dramatic flattening of the growth curve – with no further growth from the 9th till the 11th month. This could be viewed as a normalization, but it is also possible – or even probable - that it is due to loss of brain tissue, see the final MRI scan in Fig. 3. In addition to bilateral subdural fluid collection with characteristics compatible with a chronic SDH and some minor components compatible with acute rebleedings within the right frontal chronic subdural hematoma (CSDH), these CT scans also show darker areas posteriorly in the brain parenchyma that may represent imminent brain damage (white arrows) – see also Fig. 3.

This infant boy was delivered by Caesarean section at 36 + 4 weeks, originally scheduled for term but performed earlier because of premature labor. Apgar scores were 10/10/10. At the age of 13 weeks, after crying a whole night when lying on his back, difficulties lifting his head and vomiting after eating, he suddenly lost consciousness at home and became flaccid. A family member successfully performed cardiopulmonary resuscitation with return of spontaneous breathing with single, raspy breaths. On arrival at the hospital, he was unconscious, flaccid, cyanotic with marbled skin, and had irregular breathing. Fundoscopy showed numerous RH and Roth spots, and a CT scan revealed bilateral subdural fluid collections (Fig. 2). Chest X-ray was normal. After a follow-up fundoscopy on day 4, SBS was considered, and the police were notified. Retrospective analysis revealed rapid increase in head circumference (HC) (Fig. 2).

**Fig. 3:**
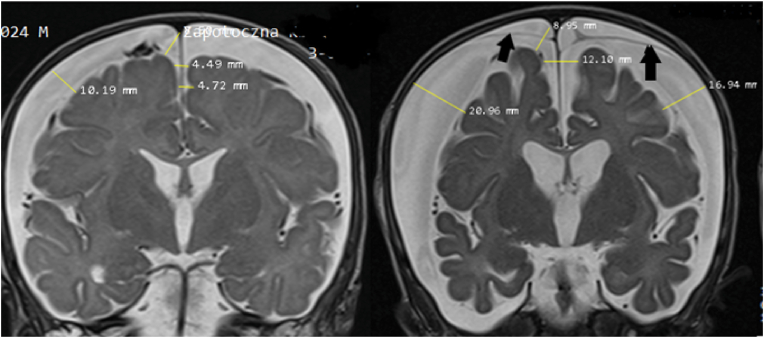
Case #2. **Left**: Coronal MRI scan at the level of Foramina Monroi 3 days after admission showing increased intracranial distances compatible with external hydrocephalus – complicated by a CSDH (Cranio-Cortical Width – CCW 10.1 mm, Sino-Cortical Width – SCW 8.5 mm, Inter-Hemispheric Width – IHW 4.7 mm) according to Lam et al. [24]. Note that the CSDH is not causing flattening of the cortical gyri or compression of the ventricles. **Right**: Coronal MRI scan at the same level five weeks later shows a dramatic increase of these intracranial distances, expansion of the fluid producing ventricles, and subdural neomembranes (black arrows) indicating that the subdural fluid collections are many weeks or even months old. The total width of the anterior horns of the lateral ventricles had increased from 26 mm to 34 mm.

A follow-up CT scan (day 3) disclosed marked increase of the extracerebral fluid collections, and MRI (day 4) confirmed the characteristics of BEH with increased intracranial distances [30]. Five weeks later, MRI showed a significant reduction in the brain volume with secondary increase of pericerebral fluid (Fig. 3). At this time, the boy and an older sister were removed from their family.

At 11 months of age, vision field loss in his right eye and a mild developmental delay were confirmed, probably caused by the long-lasting increased intracranial pressure (ICP). Flattening of the HC curve from 4 to 5 months of age may indicate minor brain atrophy, and visual impairments are possibly related to hypoxic-ischemic changes in occipital lobes. At 14 months his HC was 46.5 cm, and he could move around and walk with support. At the age of 21 months, his cognitive and motor development was adequate for his age, and he was a fully independent child with no visible limitations in his everyday life but had strabismus in his right eye.

Social services had no concerns regarding the family care, and allowed the child to be cared for by the grandparents – and in practice even by the parents themselves.

After 17 months the parents finally regained formal custody after repeated recommendations issued by the relevant child protection authority that, for the benefit of the children, they should be returned to their family. However, this decision is expected to be appealed, as criminal proceedings are still pending the opinions of the pediatricians, who have revealed no evidence against the caregivers.

***Comments***: The handling of this baby boy is remarkable and reprehensible, and not in the best interests of the children, who do best with parent-child contact, family ties being critical [31]. Prematurity [32] and male sex [33] are two well-known predisposing factors for BEH, and the extremely rapid (but clinically unnoticed) HC growth was most likely caused by raised ICP, and such an increase is nearly always due to development of hydrocephalus [29]. The predisposition for SDH in infants with BEH [34] is well-known and represents a pitfall in the diagnosis of suspected SBS [35,36].

The subdural fluid had the appearance of a chronic SDH (CSDH) with only small amounts of fresh blood, compatible with rebleeding. This can be explained by neovascularization in hematoma membranes which bleed easily [37], or by factors which interfere with normal coagulation [38]. Despite the extremely rapid HC increase over a period of several weeks (Fig. 2) and the dramatic increase of subdural fluid (Fig. 3), no surgical treatment was given to the child for subdural fluid and the increased intracranial pressure. The very suspicion of abuse may have eclipsed the need for therapeutic measures to help the child recover.

Without underpinning mechanism, RH have been considered to be "highly specific" of violent shaking or blunt force trauma [39]. Yet RH are observed in a number of other situations [25,40] and publications highlight that RH are nonspecific, secondary phenomena related to intracranial pathology [40,41]. In this case, there was no evidence for trauma, no sign of abuse or neglect, and *insufficient* evidence supporting the triad diagnostic method, including the allegedly high specificity of RH. Thus, there is no support for the default diagnosis of child abuse and ample support for interpretation as a case of BEH with secondary SDH and RH.

## 3 Discussion

Shaken Baby Syndrome (SBS) has been used for about 50 years as a medical diagnosis but was expanded into the broader and heterogeneous group of Abusive Head Trauma (AHT) in 2009) [11]. Both terms are legal accusations in a judicial context, and allegedly prove guilt in civil and criminal cases, but cause only medico-legal confusion in the accompanying court trials as well as in research [12]. Diagnostic conclusions may have the most harmful effects on the affected families, by separating parents and children. With the society's reliance on the legal system, it is of the utmost importance that any legal conclusions are based on scientific principles that allow guilt to be proven "beyond reasonable doubt" as it is phrased in criminal law in many countries. Police, prosecutors and courts lack sufficient medical knowledge, and are utterly dependent on medical expert witnesses. Therein lies the importance of medical experts, who must be impartial and base their statements on the best available scientific evidence. It is transparent that adherents of the traditional SBS hypothesis will come to radically different conclusions from experts who rests on scientific, evidence-based conclusions. The possibility arises that differences of opinion are due to value-influenced assessments among SBS proponents [7,12,13,22,23].

The unreliability of the diagnostic process in suspected SBS is amply illustrated by the ongoing controversy, by the growing literature critical of the triad diagnostic method, by the two presented cases and by other such cases [36,42]. The unreliability is further illustrated by large variations of incidence figures [43], and literature reviews [9,13] supporting this lack of reliability in basic incidence. Further, campaigns enjoining the public "do not shake a crying baby" in preventive programs, have shown no effectiveness, in that there is no indication that there is any decrease of AHT [44]. In fact, studies have shown quite the opposite. In one prevention study [45], three sham programs not associated with SBS/AHT were included. Whereas the “do not shake” program did not result in a decreased AHT incidence, surprisingly all three sham programs resulted in highly significant decreases [44]!

Furthermore, "confessions" or admissions is not a reliable way to identify true positive cases of violent shaking [25,46,47]. And the shortcomings of identifying violent shaking cases by multi-disciplinary teams, by predetermined diagnostic criteria, by court conviction, or by witnessed events, were also recently elucidated [25]. In fact, only few - if any - witnessed (or video-taped) shaken, previously healthy infants exhibit triad findings [48], allegedly signs and symptoms of AHT.

The default belief that the triad diagnostic method is reliable, when it is in fact unreliable, has repeatedly led to misdiagnosis and serious legal and social consequences [36,42] for the infant and family, augmented by delayed or wrongful treatment with permanent disabilities for the infant, as illustrated by our case #2 and others [42]. False belief and good intentions based on this unreliable triad diagnostic method, do not act in the best interests of children. There is significant risk of harm over the intended benefit, instead harming the child and its siblings. Families are divided, and an innocent parent is suspected and often convicted of a serious crime.

Despite unreliability causing collateral damage, the triad diagnostic method is still widely used, although often bolstered by the claim that "the whole picture" is somehow considered. This is contradicted in several recent publications, clinical guidelines and court cases, where reliance on the triad is heavy [49]. Moreover, if "the whole picture" is used as a diagnostic test, the only reference standard to be used is the identical test, viz. "the whole picture". But if the diagnostic test and the reference test are identical, then the result is again incorporation bias and circular reasoning - not an indicator of solid science.

Persistence of the traditional SBS hypothesis and the triad diagnostic method represent inexplicable departures from a scientific point of view. It is perhaps best understood as a belief system based on the premise that one is acting in the best interests of the child, while disregarding the obvious negative effects of this fallacious diagnostic method.

The global spread of the SBS hypothesis is not explained by its high accuracy, but rather by uncritical belief in publications and systematic reviews with basic scientific flaws in methodology, and by default dismissal of other potential medical explanations. The first case attracting worldwide publicity was the trial of British au pair Louise Woodward in Boston in 1997. This marked the first time that the underpinning of SBS was challenged by leading scientists. Despite relevant criticism, the SBS concept was vigorously defended by its proponents. Subsequent further spread of the SBS concept around the globe occurred silently, devoid of significant criticism. Despite lacking a scientific foundation, the SBS hypothesis was readily accepted and adopted in a global sociological spread. Pediatric specialties subsumed this under the moral doctrine "*First of all protect the child!*" Applying traditional SBS hypotheses, almost all triad cases will be reported as abuse, and pediatricians will not be blamed for missing a single case of violent shaking and the infant and families pay the price [36,42,50] - as in the cases presented here.

The spread of the triad diagnostic method was - and remains - uneven around the globe. Its course in Sweden [51,52] may to some extent be representative for other western countries: increased acceptance occurred during the 1990's and 2000's. A concomitant increase in infant abuse diagnoses, strongly associated with SBS criteria, culminated around and after 2010, with subsequent successive decline in incidence, in parallel with augmenting criticism of this diagnostic method [51]. Japan appears similar to Sweden, where Supreme Court decisions in 2014 and 2018 rejected the triad diagnostic method as judicially reliable per se. Detailed temporal presentations of the situation in USA, UK, France, Sweden, Japan and Australia, as well as an overview of Central and South America and countries in East Asia, can be found in a recent publication [53]. In a few countries, among others France and Norway, the existence of any controversy surrounding the SBS diagnosis was mostly ignored until 5–10 years ago, but now heated debate about the reliability of the triad diagnostic method seems to be taking place there also. The reason for the uneven acceptance of and skepticism towards the diagnostic procedure in retinodural hemorrhages is unknown, but language barriers and differences in international influence - e.g. literature, international meetings and "missionary" child abuse pediatricians, and different access to continued education may play a role. Differences in political situation and trust in public institutions, as well as media influence may also be important. Little has been published about the situation in Central and Eastern European countries. Germany seems to have a high number of alleged SBS cases, and an apparently strong adherence to the SBS hypothesis as indicated by a recent "consensus document" [54]. Unfortunately, this document is of very low scientific quality - with unreported methodology and reliance on original articles based on incorporation bias and circular reasoning. Obviously, not only France, the UK and Australia are dominated by an explicit reliance on an unproven and outdated hypothesis.

Despite a growing number of scientific reports highlighting the shortcomings and fundamental problems with medical determination of SBS, and an increasing number of acquittals and exonerations in many countries, proponents continue to double down and defend the SBS hypothesis. The acquittals and exonerations occur simultaneous with conviction, giving rise to uneven justice.

## 4 Conclusions

It is crucial that the medical investigation and treatment of a child presenting with triad findings is evidence-based. The presented cases illustrate that unsubstantiated belief in the SBS hypothesis may cause destructive medico-legal effects and increase the risk of permanent medical disabilities by depriving a child of treatment. For these reasons, the need is urgent for an open and free discussion based on sound scientific methodology. The current situation is unacceptable when all criticism of the severely flawed SBS hypothesis is rejected and wrongly dismissed as "denialism", and when incorporation bias and circular reasoning continue to represent inherent features of the systematic methodology in SBS/AHT research. Guthkelch's thoughtful statement in 2012 indeed holds true; "While controversy is a normal and necessary part of scientific discourse, there has arisen a level of emotion and divisive-ness on shaken baby syndrome/abusive head trauma that has interfered with our commit-ment to pursue the truth" [55].

## CRediT authorship contribution statement

**Anders Eriksson:** Writing – review & editing, Writing – original draft, Project administration, Formal analysis, Conceptualization. **Teresa Stachowicz-Stencel:** Writing – review & editing, Formal analysis, Data curation. **Knut Wester:** Writing – review & editing, Writing – original draft, Formal analysis, Data curation, Conceptualization.

## Patient consent statement and permission to publish imaging

Both parental couples have given written consent to publish medical data including images.

## Funding sources

This research did not receive any specific grant.

## Declaration of competing interest

The authors declare the following financial interests/personal relationships which may be considered as potential competing interests:

AE and KW have been medical experts in criminal and civil cases involving suspected child abuse. TSS has a familial and professional relationship as a professor in pediatrics to one of the cases.
