# Corrigendum to “Spread of “triad diagnostics” in suspected Shaken Baby Syndrome” [Forensic Sci. Int. Synerg. (2026) 2 100660]

**DOI:** 10.1016/j.fsisyn.2026.100676

**Published:** 2026-04-03

**Authors:** A. Eriksson, T. Stachowicz-Stencel, K. Wester

**Affiliations:** a Department of Clinical Sciences, Forensic Medicine, Umeå University, Sweden; b Department of Pediatrics, Hematology and Oncology, Medical University of Gdansk, Poland; c Department of Clinical Medicine, University of Bergen, Norway

The authors regret incorrect numbering of references in the captions of Figs. 2 and 3. In Fig. 2, the correct reference is no. 29, and in Fig. 3, the correct reference is no. 30.

The authors would like to apologise for any inconvenience caused.

